# Editorial: Developmental dysplasia of the hip in children: The role of early diagnosis and treatment

**DOI:** 10.3389/fped.2023.1138999

**Published:** 2023-02-03

**Authors:** Thomas Baumann, Stefan Essig

**Affiliations:** ^1^ Independent Researcher, Solothurn, Switzerland; ^2^ Department of Health Sciences and Medicine, University of Lucerne, Lucerne, Switzerland

**Keywords:** newborn, hip ultrasound, orthosis, surgery, developmental dysplasia of the hip (DDH)

Editorial on the Research Topic Developmental dysplasia of the hip in children: The role of early diagnosis and treatment

Developmental dysplasia of the hip (DDH) is one of newborns’ most common causes of musculoskeletal health conditions. Our Research Topic, “Developmental Dysplasia of the Hip in Children: The Role of Early Diagnosis and Treatment”, provides insight into DDH diagnosis and treatment aspects.

A diagnosis of DDH in the first weeks of life should be made by ultrasound. Graf established ultrasound examinations of the hip of the newborn (1), but other authors such as Harcke (2), Terjesen (3), Suzuki (4) and Morin (5) have also promoted ultrasound methods. In addition, there are adaptations of the Graf system by Rosendahl (6) and Baumann (7). The most widely used way is that of Graf. However, the problematic comparability of results between the diverse methods has severely hampered its dissemination. Ossendorff et al. describe in their article user errors and insufficient application of the Graf quality assessment algorithm as other causes. Furthermore, as Walter et al. report, the quality of published articles on hip examinations that follow the Graf approach is often insufficient. Therefore, the discussion about the reliability of diagnostic methods rightly continues. One question that is also intensely debated in this context is whether a general screening or an examination according to risk or clinical findings is beneficial. Here, the work of Han and Li presents an argumentative basis for a universal ultrasound screening to prevent late-detected cases.

There are many uncertainties not only in diagnostics but also in therapy. Although many authors assume that treatment should start as early as possible, there are still significant differences of opinion on the “how” (8). Studies show a largely “eminence-based” rather than “evidence-based” approach. Thus, conservative as well as surgical procedures are used. There has yet to be an international consensus to optimise and simplify the therapy according to scientific aspects. The paper by Chaibi et al. concerns the issue of the reliability of the Tübingen splint as a treatment option for unstable hips. Gou et al. have presented an interesting paper regarding the use of the Pavlik harness.

In our Research Topic, insightful papers could be published quickly. We hope to see more publications that shed light on the early diagnosis and therapy of DDH to optimise its management and improve the prognosis for the affected children.

## Author contributions

All authors listed have made a substantial, direct, and intellectual contribution to the work and approved it for publication.
